# Associations of Air Pollution and Pediatric Asthma in Cleveland, Ohio

**DOI:** 10.1155/2021/8881390

**Published:** 2021-09-15

**Authors:** Sumita B. Khatri, Cynthia Newman, Jeffrey P. Hammel, Tanujit Dey, Jeffrey J. Van Laere, Kristie A. Ross, Jerri A. Rose, Timothy Anderson, Shaibal Mukerjee, Luther Smith, Matthew S. Landis, Ann Holstein, Gary A. Norris

**Affiliations:** ^1^Cleveland Clinic, 9500 Euclid Ave/A90, Cleveland, OH, USA; ^2^Case Western Reserve University School of Medicine, Cleveland, OH, USA; ^3^MetroHealth Medical Center, 2500 Metrohealth Dr, Cleveland, OH 44109, USA; ^4^Brigham and Women's Hospital, Harvard Medical School, Boston MA, USA; ^5^Henry Ford Hospital, Johns Hopkins School of Medicine, 733 N Broadway, Baltimore, MD 21205, USA; ^6^Rainbow Babies and Children's Hospital, 11100 Euclid Ave, Cleveland, OH 44106, USA; ^7^Beth Israel Deaconess Medical Center, 330 Brookline Ave, Boston, MA 02215, USA; ^8^U.S. Environmental Protection Agency Office of Research and Development, Raleigh, NC, USA; ^9^Serco Inc., 12930 Worldgate Dr No. 600, Herndon, VA 20170, USA; ^10^Chagrin Valley Engineering, Ltd., 22999 Forbes Rd STE B, Cleveland, OH 44146, USA

## Abstract

Air pollution has been associated with poor health outcomes and continues to be a risk factor for respiratory health in children. While higher particulate matter (PM) levels are associated with increased frequency of symptoms, lower lung function, and increase airway inflammation from asthma, the precise composition of the particles that are more highly associated with poor health outcomes or healthcare utilization are not fully elucidated. PM is measured quantifiably by current air pollution monitoring systems. To better determine sources of PM and speciation of such sources, a particulate matter (PM) source apportionment study, the Cleveland Multiple Air Pollutant Study (CMAPS), was conducted in Cleveland, Ohio, in 2009–2010, which allowed more refined assessment of associations with health outcomes. This article presents an evaluation of short-term (daily) and long-term associations between motor vehicle and industrial air pollution components and pediatric asthma emergency department (ED) visits by evaluating two sets of air quality data with healthcare utilization for pediatric asthma. Exposure estimates were developed using land use regression models for long-term exposures for nitrogen dioxide (NO_2_) and coarse (i.e., with aerodynamic diameters between 2.5 and 10 *μ*m) particulate matter (PM) and the US EPA Positive Matrix Factorization receptor model for short-term exposures to fine (<2.5 *μ*m) and coarse PM components. Exposure metrics from these two approaches were used in asthma ED visit prevalence and time series analyses to investigate seasonal-averaged short- and long-term impacts of both motor vehicles and industry emissions. Increased pediatric asthma ED visits were found for LUR coarse PM and NO_2_ estimates, which were primarily contributed by motor vehicles. Consistent, statistically significant associations with pediatric asthma visits were observed, with short-term exposures to components of fine and coarse iron PM associated with steel production. Our study is the first to combine spatial and time series analysis of ED visits for asthma using the same periods and shows that PM related to motor vehicle emissions and iron/steel production are associated with increased pediatric asthma visits.

## 1. Introduction

Air pollution has been associated with poor health outcomes and continues to be a risk factor for respiratory health. Epidemiologic, panel, and longitudinal cohort studies in adults and children have demonstrated increased frequency of asthma symptoms, new onset of asthma, worsening of lung function, reduced pace of lung development, and increased airway inflammation, including from oxidant stress [1–4]. During times of poor air quality with higher levels of air pollution, the deleterious effects are evident by acute and chronic symptomatology and development of chronic diseases, such as allergic rhinitis and asthma [5–13]. Ironically, these clinical effects occur even when PM levels are within air quality guidelines [14, 15]. Associations between adverse human health outcomes and ambient particulate matter (PM) concentrations that are attributed to emissions from motor vehicles, coal-fired power plants, iron and steel production, residual oil combustion, and resuspended dust have been reported [5, 9, 10, 13]. Multiple components of air pollution, such as elemental carbon, nitrogen dioxide (NO_2_), sulfur dioxide (SO_2_), nickel, and vanadium, have been associated with cardiovascular- and respiratory-related hospitalizations [6, 9, 11, 12, 16]. Furthermore, inorganic components of PM have been associated with cardiovascular- and respiratory-related hospitalizations and attributed to air pollution from motor vehicles (elemental carbon, NO_2_), oil combustion (nickel, vanadium), and secondarily formed aerosols from coal combustion-emitted sulfur dioxide [10, 17–19].

As adults may be exposed to a variety of chronic exposures and develop chronic health conditions, epidemiologic studies of children and their onset of new or worsening symptoms of acute or chronic diseases (allergic rhinitis, asthma) provide a specific and compelling rationale to improve air quality in a wholescale manner. Scientific evidence as noted suggest that a combination of local and regional sources may be having an impact on children's health, particularly with respect to asthma [10]. All these data suggest that a combination of local sources in the Cleveland airshed and regional sources outside the airshed may be having an impact on children's health, modified further by weather, wind speed and direction, and geographic factors.

Air pollution, including from steel mills, has been shown to affect children's health adversely, particularly as related to problems such as bronchitis and asthma, and traffic-related air pollution is also a source of childhood morbidity [2, 9, 15, 19, 20]. This current study, Cleveland Multiple Air Pollutant Study (CMAPS) in Cleveland, OH, was designed to evaluate sources contributing to fine PM (also known as PM_2.5_; PM < 2.5 *μ*m aerodynamic diameter) and coarse PM (aerodynamic diameter between 2.5 and 10 *μ*m), using a combination of high time resolution and spatial measurements [8, 21–27]. Cleveland was selected due the complex mixture of PM sources and the plausible significant respiratory health associations that may be revealed [14, 28, 29]. A recently published CMAPS toxicology study of nonallergic and dust mite–allergic Balb/cJ mice reported significant responses for coarse PM components from both urban and rural sites in Northeast Ohio, which underscores the need to study not only the epidemiology of such associations but also the molecular mechanisms and pathobiology, which account for the underlying the associations of worsening airways diseases [30–33].

This current article presents a pediatric health study that employed CMAPS-measured and modeled data to evaluate the association of air pollution source categories (referred to here as components) with pediatric asthma emergency department (ED) visits. Both long-term measures of air pollution via spatial analysis and short-term (daily) components contributing to observed PM and meteorological conditions were used as the exposures of concern.

## 2. Methods

### 2.1. Ambient Air Measurements and Models

**Figure 1:**
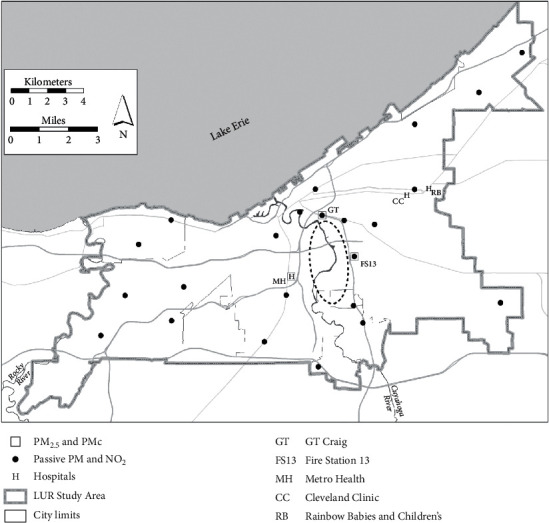
Locations of participating Cleveland hospitals (CC = Cleveland Clinic, RB = University Hospitals Rainbow Babies, and MH = Metro Health), monitoring sites, and the industrial valley located within the dashed line.

CMAPS land use regression models (LUR) for predicting NO_2_ and coarse PM at patients' home addresses were developed using passive air samplers at spatially representative sites (Figure 1) during Summer 2009 and Winter 2010 intensives (periods of intense detailed monitoring periods), with predictor variables, such as traffic, point source, and area emission sources. As presented elsewhere, LUR predictions revealed elevated concentrations of NO_2_ and PM near major roadways and the industrial valley area south of downtown Cleveland, suggesting a priori that nonuniform exposures exist [23]. Summer and winter LUR-predicted concentrations were averaged to represent long-term exposure, and the average concentration was associated with a patient's residence.

**Figure 2:**
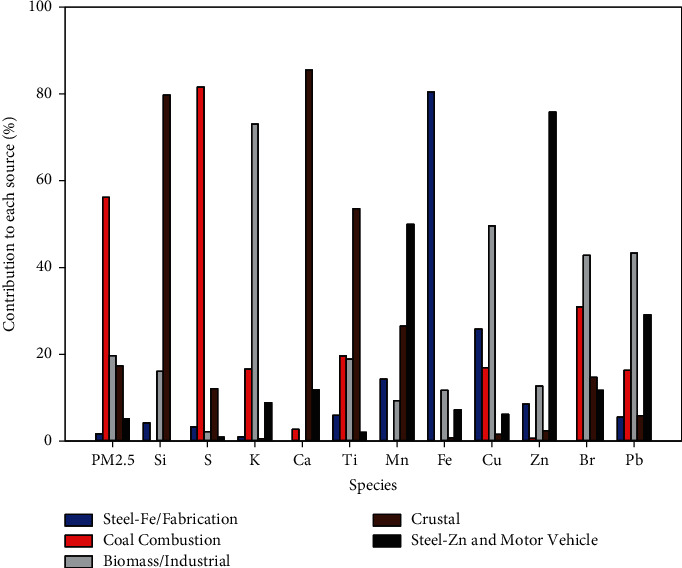
Fine PM component source profiles.

**Figure 3:**
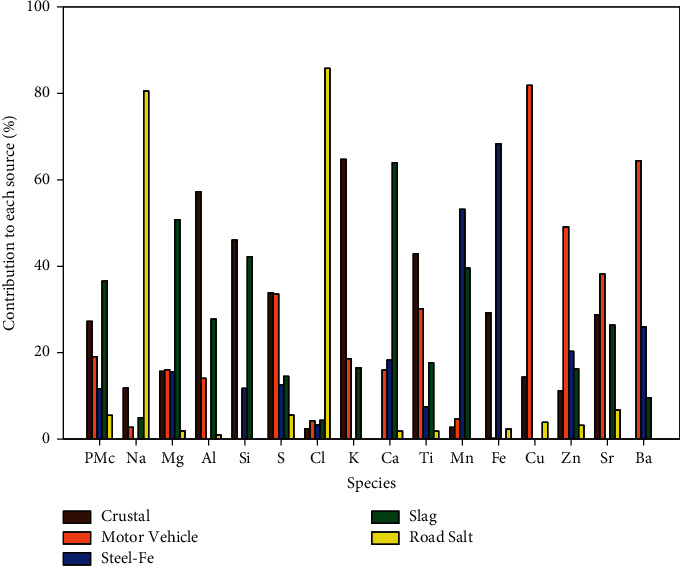
Coarse PM source profiles.

Daily fine and coarse PM filter measurements were collected at two sites, GT Craig and Metro Health (Figure 1), using sequential dichotomous samplers and then analyzed for element species with energy dispersive X-ray fluorescence [34, 35]. Receptor modeling was used with the daily PM measurements to estimate short-term local speciated components of air pollution, after which associations with pediatric asthma ED visits were evaluated [13]. The US EPA's Positive Matrix Factorization (EPA PMF 5.1) model determined fine and coarse PM contributions from air pollution sources [36]. Contributions from two daily 12-hour measurements and two sites were averaged each day. Five fine PM components and their indicator elements were identified; the percent contribution to each component is shown in Figures 2 and 3: industrial and biomass burning (K, Cu, Br, and Pb), crustal (Si, Ca, and Ti), coal combustion (S), iron/steel production and fabrication (Fe), and steel production and motor vehicle (Mn and Zn). Five coarse PM components were identified (Figure 3): road salt (Na and Cl), slag and sintering (Mg, Ca, and Mn), iron/steel production and fabrication (Fe), crustal (Al, Si, K, and Ti), and motor vehicle and resuspended road dust (S, Cu, Zn, and Ba) [22, 37–40]. Microscopy analyses also found elevated concentrations of iron particles from high-temperature steel production and fabrication processes in Cleveland [21].

### 2.2. Health Data

Data on daily pediatric emergency department (ED) visits for acute asthma were collected from three local pediatric hospitals from August 1, 2009, to July 31, 2010 (Metro Health Medical Center, Cleveland Clinic, and Rainbow Babies and Children's). After IRB approval at each institution, date of visit, age, gender, and home address of children aged 2–17 years who visited the ED with a primary diagnosis of asthma were collected. Home addresses of patients were mapped with ArcGIS® software; 85% were mapped successfully.

### 2.3. Health Outcomes and Epidemiological Models

The first phase of analysis included an evaluation on the prevalence of population-adjusted asthma ED visits related to long-term exposures from LUR. A combined long-term and daily parameter analysis with Poisson regression modeling using population-adjusted ED visits as outcome was then performed, with speciated daily levels or mass levels of PM pollution as independent variables. Next, a separate model was constructed for each PM component or mass level to evaluate short-term pollution contribution to the long-term spatial model (LUR). The model for each PM component or mass level contained adjustments for LUR quartile of NO_2_ (and separate model runs for the coarse PM LUR quartiles); daily median mixing height (a measure of atmospheric boundary layer); daily minimum temperature; and a categorical variable for season (spring/summer, fall, or winter). In each model, speciated PM components or mass levels, and mixing height, were log_2_-transformed to eliminate the impact of skewness in their distribution; a cubic spline of minimum temperature was used. The model is represented as follows:

(1) $$\begin{array}{c} Y=\beta_{0}+\beta_{1}\log_{2}\left(X\right)+\beta_{2}\log_{2}\left(\text{MH}\right)+\beta_{3}\left(\text{LUR}\_2Q\right)+\beta_{4}\left(\text{LUR}\_3Q\right)\\ +\beta_{5}\left(\text{LUR}\_4Q\right)+\beta_{6}\left(S\_\text{FALL}\right)+\beta_{7}\left(S\_\text{WINTER}\right)+\left(\text{SPF}\_\min\text{temp}\right), \end{array}$$

where *Y* = population-adjusted ED visits; *X* = PM component or mass level; MH = median daily mixing height; LUR_2*Q*, LUR_3*Q*, and LUR_4*Q* = second, third, and fourth LUR NO_2_ or coarse PM quartile, respectively (NO_2_ and coarse PM quartiles run in separate models); S_FALL = fall (observation between 9/22/2009 and 12/20/2009); S_WINTER = winter (observation between 12/21/2009 and 3/15/2010); and SPF (min temp) = cubic spline function of minimum daily temperature.

Models were repeated with moving-average lag 3-day and lag 7-day values (that is, averages over the period from the current day to 3 or 7 days prior) applied to mixing height, minimum temperature, and speciated PM levels. For each PM component or mass level evaluated by the Poisson regression model, a significance level of 0.005 was used to assess its association with population-adjusted ED visits. This Bonferroni-adjusted significance level ensured an overall significance level of 0.05 among the separate models for all 10 PM components. A Bonferroni correction was not used to evaluate significance of the LUR quartile, mixing height, and minimum temperature because the same data were used for each PM component model. Models were repeated with a squared term for PM components (*β*_8_(log_2_(*X*))^2^) to assess evidence of nonlinearity with respect to log_2_-transformed PM with minimal allocation of degrees of freedom.

## 3. Results

Pediatric asthma-related ED visit data were collected between August 1, 2009, and July 31, 2010; 6180 episodes of asthma-related visits for children aged 2–17 years were collected. Of this group, geographic matching was possible for 5868 (65%), and of these patients, 1837 lived in the area with exposure estimates that could be linked to their street address of record. Approximately 76% experienced one ED visit during that time, and 24% had two or more visits in that period.

Exposure-response relationships of long-term NO_2_ and PM coarse levels determined by LUR methodology and population-adjusted prevalence of ED visits for asthma in children are shown in Table 1. Similar to LUR-based long-term air quality levels of coarse PM and NO_2_ quartiles, univariate analysis of mixing height (daily median) alone found that the prevalence of ED visits was 2.02/100,000 when mixing height was lower (<250 m) and 1.58/100,000 when mixing height was higher (500–750 m) (Table 2).

Poisson regression modeling analysis was performed using combined LUR quartiles and daily exposure of PM source contributions (speciated levels), daily minimum temperature, season, and mixing height. The number of days ranged from 147 to 161 in the multivariable modeling because some days did not have complete median mixing height and PM data. Results in terms of the estimated means ratio of pediatric asthma visits, based on a doubling of the predictor variables, are reported in Table 3. The means ratio represents proportional change in expected ED visits, with a value of 1.0 meaning “no change.” For example, Table 3 indicates that modeling estimates show that ED visits would increase by 11.4% if the fine steel Fe/fabrication component were doubled, and visits would decrease between 11.6% and 13.9% with a doubling of mixing height.

PM associated with steel production demonstrated significant associations with pediatric asthma visits, particularly with respect to fine and coarse PM steel Fe and fabrication components. The composite industrial fine PM component of Br, K, and Pb was not significantly associated with pediatric ED visits for asthma; neither was the element S, the predominant identifying species of coal-fired power plants (Figure 2), nor the crustal element component representing fine PM soil fraction. Neither coarse nor fine PM mass was statistically significant as a predictor of pediatric ED visits for asthma, an independent variable, when evaluated in their own Poisson regression models (*p*=0.11 and *p*=0.74, respectively). In analyses based on the application of 3-day or 7-day lags to daily measurements, increased speciated PM components did not correspond to increased ED visits with statistical significance, except for increased 7-day average of Fine steel Fe/fabrication, with a covariate-adjusted means ratio (95% CI) of 1.173 (1.093–1.260) (*p* < 0.0001) per doubling. This was greater, but not statistically different, compared with the estimated means ratio resulting from the current-day model for Fine steel Fe/fabrication (1.114 (1.072–1.153)). In current-day models, none of the subsequently tested squared terms for PM components were significant at the Bonferroni-corrected 0.005 significance level, when added to the models, so there was no convincing evidence that log_2_ transformations yielded nonlinear relationships. Furthermore, the log_2_ transformations eliminated skewness in distributions of PM component measures, alleviating undue influence of some of the highest measurements.

The LUR estimate-related associations are indicators of motor vehicles [23]. In addition, NO_2_ was found to be associated with vehicular exhaust, and coarse PM from resuspended road dust and tire wear [39]. With respect to speciated PM and mixing height, model inferences did not change with the use of coarse PM LUR quartiles, compared with NO_2_ quartiles because the model parameter estimation for these daily measures was driven completely by total ED visits per day. Minimum daily temperature and indicators for seasonality were not significant in most models, although they were in some cases (Table 3). However, due to the collinear relationship between season and low temperature, it not possible to disentangle these different effects from each other. The results obtained here suggest a potential effect from these variables, either individually or possibly in combination. But both were retained in the models so that associations between ED visits and PM components could account for temperature and seasonality. Relative to the 1st quartile, LUR 3rd and 4th quartiles were associated with increased ED visits for both coarse PM and NO_2_ (Tables 1 and 3).

## 4. Discussion and Conclusion

This study capitalized on the unique opportunity to evaluate associations between pediatric ED visits for asthma and both long- and short-term exposures to air pollutants, using measurement data collected during both periods. From a long-term exposure standpoint, our study demonstrates that children living in areas experiencing higher quartiles of air pollution had a higher mean number of visits: a nearly two-fold greater prevalence of asthma visits was seen at the highest versus lowest quartile of coarse PM and NO_2_ levels. Our modeling indicated that lower atmospheric boundary layer mixing heights led to increased ED visits. Because atmospheric dispersion decreases with lower boundary layer mixing heights [41], the model suggests that undiffused or concentrated local air pollution emissions are relevant.

A major strength of our study design is that it also evaluated the relationship of PM components (i.e., species-specific fine and coarse PM) with a specific respiratory disease in children [11]. Our methodology to evaluate childhood asthma only (and not include adult asthma) mitigated confounding conditions that are related to older age, such as smoking and other chronic conditions (such as COPD and heart disease), which might have contributed to misclassification and affect the associations clearly noted in our study of pediatric asthma. Prior studies have demonstrated the strong association of PM_10_ with asthma, even in the presence of carbon monoxide (CO) and NO_2_, with motor vehicle sources as the predominant factor when also taking into account secondary sources such as sulfates and resuspended soil [6, 12]. Our findings agree with others that demonstrated associations of traffic-related pollutants such as NO_2_ [19]. Many studies have also found that long-term exposure to air pollution plays a role in the occurrence of adverse health effects, particularly in children's health, where ozone and PM pollutants are associated with lower rates of lung growth and incidence of asthma [42, 43]. Our research underscores these findings.

Steel production has been found to be associated with health effects in the Utah Valley Study [9] and in the Netherlands [44]. This study demonstrates that ambient iron-containing PM emitted from local steel production was associated with increased ED asthma visits in Cleveland. Iron is a crustal element component of fine and coarse PM commonly associated with resuspended or wind-blown dust [45] and has been shown to have significant associations with asthma symptoms and increased ED asthma visits [46]. The components of iron contributing to the PM at sites south of downtown Cleveland were characterized, and the main source was emissions from iron/steel production, with the highest concentrations in the industrial valley south of downtown [23, 27]. Particle size distribution for Cleveland iron-containing particles showed that the iron contributed to both fine and coarse PM, with most particles being >2.5 *μ*m in median aerodynamic diameter [21].

Steel production is also a source of zinc, which can be contributed by sources such as motor vehicles as well [39]. Studies not located near steel production have also demonstrated that childhood asthma was associated with elemental carbon and zinc from motor vehicles [47, 48]. Sinclair et al. found that zinc was significantly associated with childhood asthma outpatient visits in Atlanta, GA [48]. From the standpoint of short-term or daily variability of pollutant exposure, however, our study did not find a statistically significant association with zinc source components; this may be due to significant local point sources of zinc in Cleveland.

We found that short-term coarse PM motor vehicle and fine PM crustal-related pollution were not significantly associated with pediatric ED asthma visits (respective *p* values of 0.009 and 0.008, compared with Bonferroni-adjusted significance level of 0.005 to an overall significance level of 0.05); however, an association is both clinically and scientifically plausible. Coarse motor vehicle and fine crustal components are mixtures of road dust and crustal material, which contains historical industrial and motor vehicle PM. Furthermore, LUR-estimated long-term exposures to NO_2_ and coarse PM had significant associations, suggesting that longer-term regional or near-road impact is more important than daily variability.

While the methodology and speciation of PM pollution presented in our study is a unique strength, there are limitations to this study, which may confound the results. With our retrospective epidemiologic study, the ability to collect information related to indoor air quality (including exposure to environmental tobacco smoke exposure) and certain components of patient and family history was not consistently available. In addition, our outcome, which was a pediatric ED visit for asthma, could not be further refined in the context of the asthma severity of the patient nor whether any additional visits to outpatient care had occurred for asthma exacerbations.

Key findings and conclusions in this study came from evaluating health impacts using both short- and long-term exposure estimates, and the breakout of fine and coarse PM pollution into probable source components. The failure to find statistically significant relationships between each of coarse and fine PM mass measurements and pediatric ED visits for asthma in the Poisson regression modeling emphasizes the benefit of investigating individual source components. This approach found (i) a significant association with pediatric asthma visits and short-term exposures to the iron PM component associated with steel production (even with a potentially greater uncertainty in the analysis due to temporary shut-down of select steel plant operations during the winter period of our study) and (ii) higher frequency of ED asthma visits with increasing LUR coarse PM and NO_2_ estimates, which are primarily contributed by motor vehicles. From this collaborative air pollution and public health study, we report the first combined spatial and temporal analysis of ED visits for asthma and demonstrate that motor vehicle emissions (NO_2_) and local metal production/fabrication are associated with increased pediatric asthma visits.

## Figures and Tables

**Table 1:** Prevalence of population-adjusted pediatric emergency department visits for asthma, based on home location-based air quality levels.

LUR subset	Mean number of ED visits/100,000 people
Coarse PM Q1 (0–6.4 *μ*g m^−3^)	1.46
Coarse PM Q2 (6.4–7.8 *μ*g m^−3^)	1.25
Coarse PM Q3 (7.8–9.6 *μ*g m^−3^)	1.96
Coarse PM Q4 (9.6–22 *μ*g m^−3^)	2.62
NO_2_ Q1 (3.0–13 ppb)	1.16
NO_2_ Q2 (14–15 ppb)	1.47
NO_2_ Q3 (15–17 ppb)	2.16
NO_2_ Q4 (17–27 ppb)	2.22

**Table 2:** Lower median daily mixing height associated with greater proportion of population-adjusted pediatric emergency department visits for asthma.

Mixing height (current day)	Mean number of ED visits/100,000 people
<250 m	2.02
250–500 m	1.68
500–750 m	1.58
>750 m	1.17

**Table 3:** Poisson regression models for each of the fine and coarse PM components individually, adjusting for daily minimum temperature, daily median mixing height, and NO_2_ LUR quartiles.

Component	Average of Metro Health & GT Craig sites	
	MR^a^ (95% CI) per doubling	*p* value^b^
Fine biomass/industrial	1.028 (0.968–1.091)	0.37
Fine steel Zn and motor vehicle	0.992 (0.954–1.032)	0.69
Fine crustal	1.084 (1.020–1.153)	0.009
Fine steel Fe/fabrication	1.114 (1.072–1.153)	**<0.001**
Fine coal combustion	0.995 (0.952–1.041)	0.84
Coarse road salt	1.008 (0.991–1.025)	0.35
Coarse motor vehicle	1.072 (01.018–1.129)	0.008
Coarse slag and fly-ash	0.999 (0.973–1.024)	0.91
Coarse crustal	1.051 (0.995–1.111)	0.08
Coarse iron/steel production and fabrication	1.064 (1.029–1.100)	**<0.001**

Results over the 10 individual PM component models		
Minimum temperature^c^		0.043 to 0.26
Mixing height^d^	0.861 (0.782–0.949) to 0.884 (0.801–0.977)	**≤0.016**
Seasonality^e^ fall	1.13 (0.94–1.36) to 1.26 (1.05–1.51)	0.013 to 0.18
Winter	0.93 (0.71–1.23) to 1.07 (0.83–1.38)	≥0.62

LUR NO_2_ quartile^f^		**<0.001**
1^st^	1	
2^nd^	1.316 (1.133–1.528)	
3^rd^	1.922 (1.654–2.234)	
4^th^	1.893 (1.589–2.256)	

LUR coarse PM quartile^g^	1	**<0.001**
1^st^	0.898 (0.777–1.038)	
2^nd^	1.314 (1.140–1.514)	
3^rd^	1.789 (1.517–2.109)	
4^th^		

^a^MR: means ratio = proportional change in the mean number of population-adjusted ED visits per day. ^b^*p* values are summarized across the models for the different pollution components. ^c^Minimum temperature: no MR estimates are calculated because temperature is modeled using smoothing splines. ^d^Mixing height: results are summarized as a range across the models for the different pollution components. ^e^Seasonality: MR corresponds to a comparison to spring/summer. ^f^LUR quartiles: MR corresponds to a comparison to the 1^st^ quartile. Results are summarized for the component models with the most available data. ^g^Results reported for model constructed with coarse PM quartiles in place of NO_2_ quartiles. The bold p values reflect statistical significance in our model and meet the rigor of Bonferroni-corrected 0.005 significance level.

## Data Availability

The data used to support the findings of this study are available at US Environmental Protection Agency Office of Research and Development.
